# A component of high‐grade fetal lung adenocarcinoma diagnosed as the cause of lymph node metastasis

**DOI:** 10.1111/1759-7714.15296

**Published:** 2024-03-25

**Authors:** Daichi Ishii, Satoshi Aoyama, Wataru Arai, Masaru Hasegawa, Akihiko Tanaka, Motoki Sakuraba

**Affiliations:** ^1^ Department of Thoracic Surgery Sapporo City General Hospital Sapporo Japan; ^2^ Department of Pathology Sapporo City General Hospital Sapporo Japan; ^3^ Department of Respiratory Medicine Sapporo City General Hospital Sapporo Japan

**Keywords:** high‐grade fetal lung adenocarcinoma, lung cancer, lymph node metastasis, thyroid transcription factor‐1

## Abstract

High‐grade fetal lung adenocarcinoma (H‐FLAC) is a rare type of tumor. There have been no reports demonstrating the degree of metastatic susceptibility of this tumor type. In this report, we describe a case in which 15% of the adenocarcinoma components were H‐FLAC diagnosed as the cause of lymph node metastasis. A 75‐year‐old man presented with suspected primary lung cancer (clinical stage IIA, T2bN0M0) and underwent left upper lobectomy and superior mediastinal lymph node dissection. Postoperative histopathology revealed lung cancer with only lobar bronchial lymph node (#11) metastasis. Approximately 60% of the invasive adenocarcinoma showed a papillary morphology, 25% showed a lepidic morphology, and 15% showed a fetal morphology. The histomorphological and immunohistological features of #11 metastasis were similar to those of H‐FLAC. Herein, we report a rare and important case of H‐FLAC with proven lymph node metastasis, showing that even a small amount of H‐FLAC tissue can cause metastasis.

## Graphical abstract

This is the first report to demonstrate the histological characteristics of high‐grade fetal lung adenocarcinoma (H‐FLAC) as the cause of N1 in a patient with lung adenocarcinoma containing a small number of H‐FLAC components.

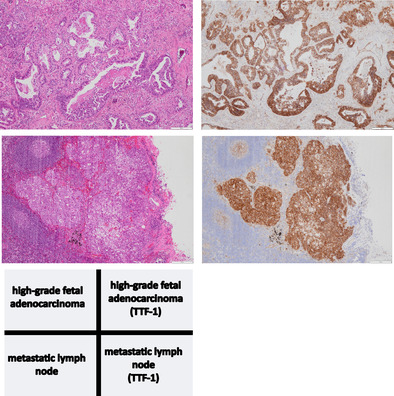

## INTRODUCTION

Fetal lung adenocarcinoma, classified as a special type of adenocarcinoma according to the 2004 World Health Organization classification, is a rare tumor composed of components similar to those of the fetal lung during the adenoid stage (5–16 weeks).^1^ Adenocarcinomas with a partial component showing high‐grade histology account for approximately 0.4% of all lung cancers.^2^ Approximately 60% of fetal lung adenocarcinomas are reportedly stage II or higher at diagnosis, and most are detected in advanced stages with a poor prognosis.^2^ However, there are no reports on the degree of metastatic susceptibility of these tumor types. We describe a case in which 15% of the adenocarcinoma components had the histological features of high‐grade fetal lung adenocarcinoma (H‐FLAC), which proved to be the cause of N1.

## CASE REPORT

**FIGURE 1:**
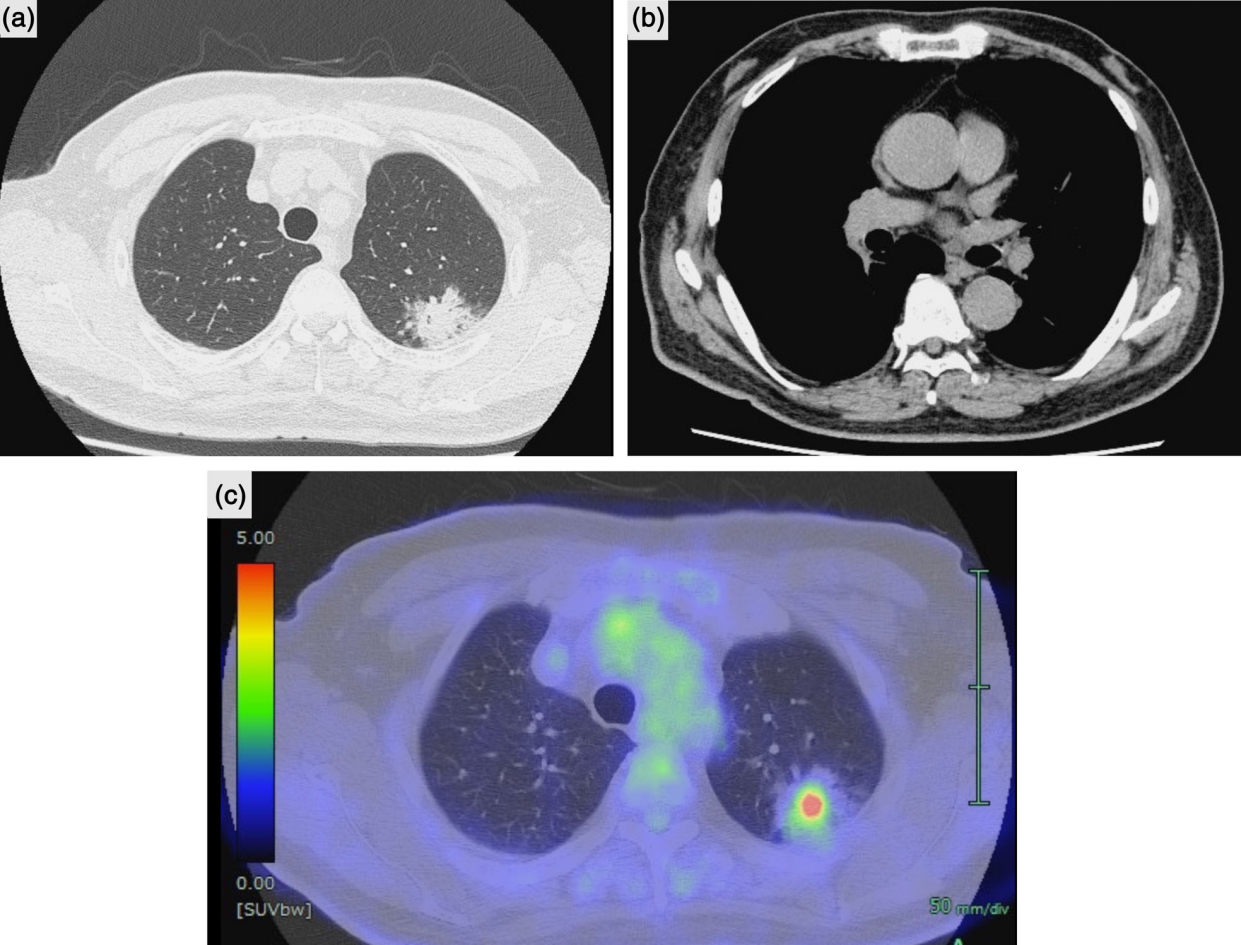
(a, b) Preoperative chest computed tomography. Computed tomography shows a 43‐mm nodule at the periphery of S^1+2^ of the left upper lobe. There is no obvious lymph node enlargement. (c) The maximum standard uptake value of the positron emission tomography is 7.5.

A 75‐year‐old man presented with suspected primary lung cancer (clinical stage IIA, T2bN0M0) in his left upper lobe. His medical history included cholelithiasis, hypertension, and colorectal polyps. Physical findings were unremarkable. The serum carcinoembryonic antigen (CEA) level was 11.2 (normal range 0–5.0) ng/mL, and the carbohydrate antigen 19–9 (CA19‐9) level was 44 (normal range 0–37) U/mL, although other laboratory test findings were unremarkable. Spirometry test results were normal. Computed tomography (CT) showed a 43‐mm nodule at the periphery of S^1 + 2^ of the left upper lobe (Figure 1a). No obvious lymph node enlargement was observed on CT (Figure 1b). The maximum standard uptake value on positron emission tomography was 7.5, and there were no findings suggestive of obvious metastasis. (Figure 1c). Left upper lobectomy and superior mediastinal lymph node dissection were performed. The operative time was 187 min and blood loss volume was 20 mL. Postoperative histopathology revealed double lung cancer with only lobar bronchial lymph node (#11) metastasis (invasive adenocarcinoma [invasive size is 31 mm, pT2aN1M0 stage IIB] and adenocarcinoma in situ [overall diameter 4 mm, pTis]). Approximately 60% of the invasive adenocarcinoma showed a papillary morphology, 25% showed a lepidic, and 15% showed a fetal morphology.

**FIGURE 2:**
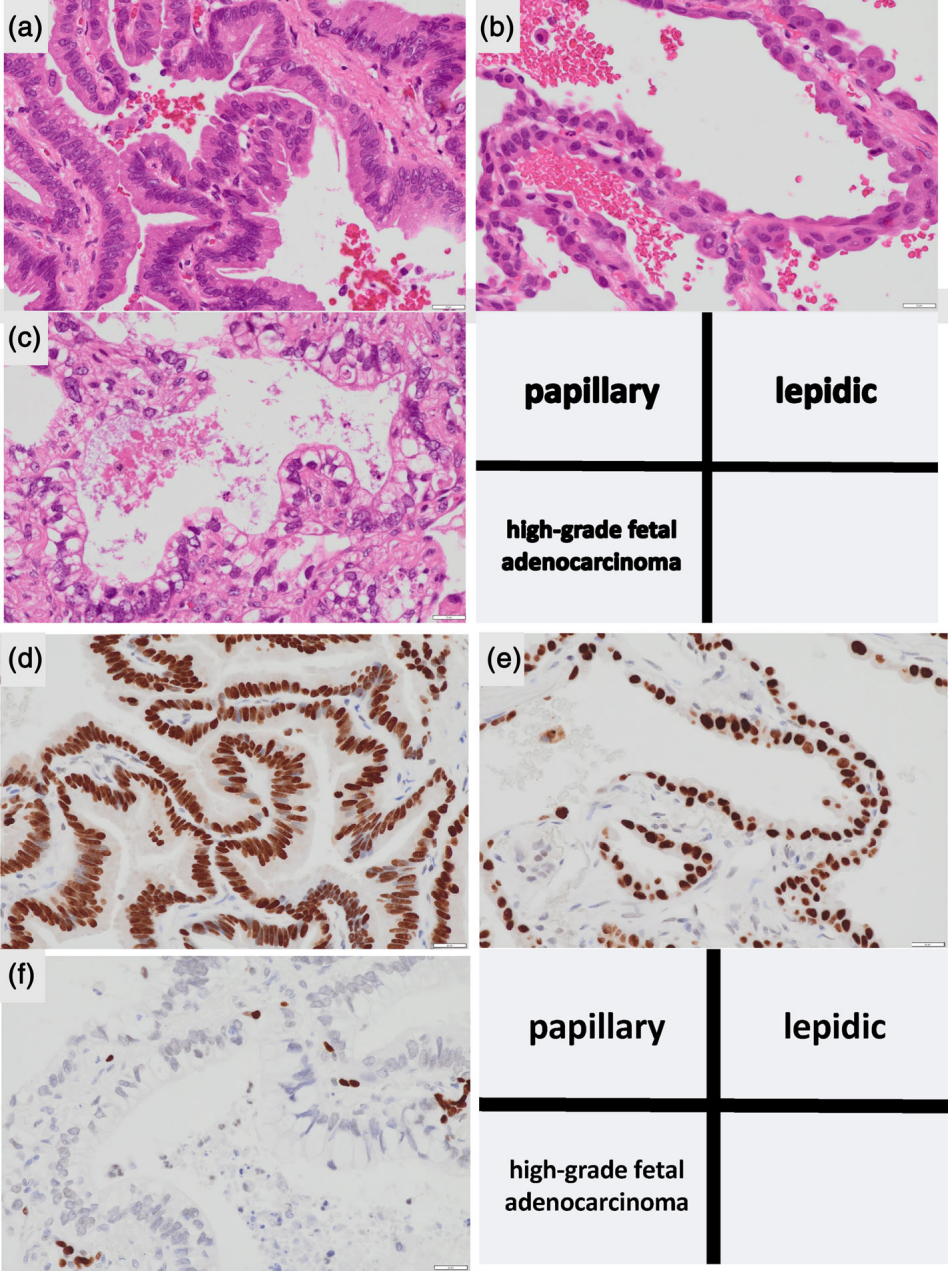
(a–c) Microscopic findings. (d–f) Special and immunohistochemical stains for thyroid transcription factor‐1.

**FIGURE 3:**
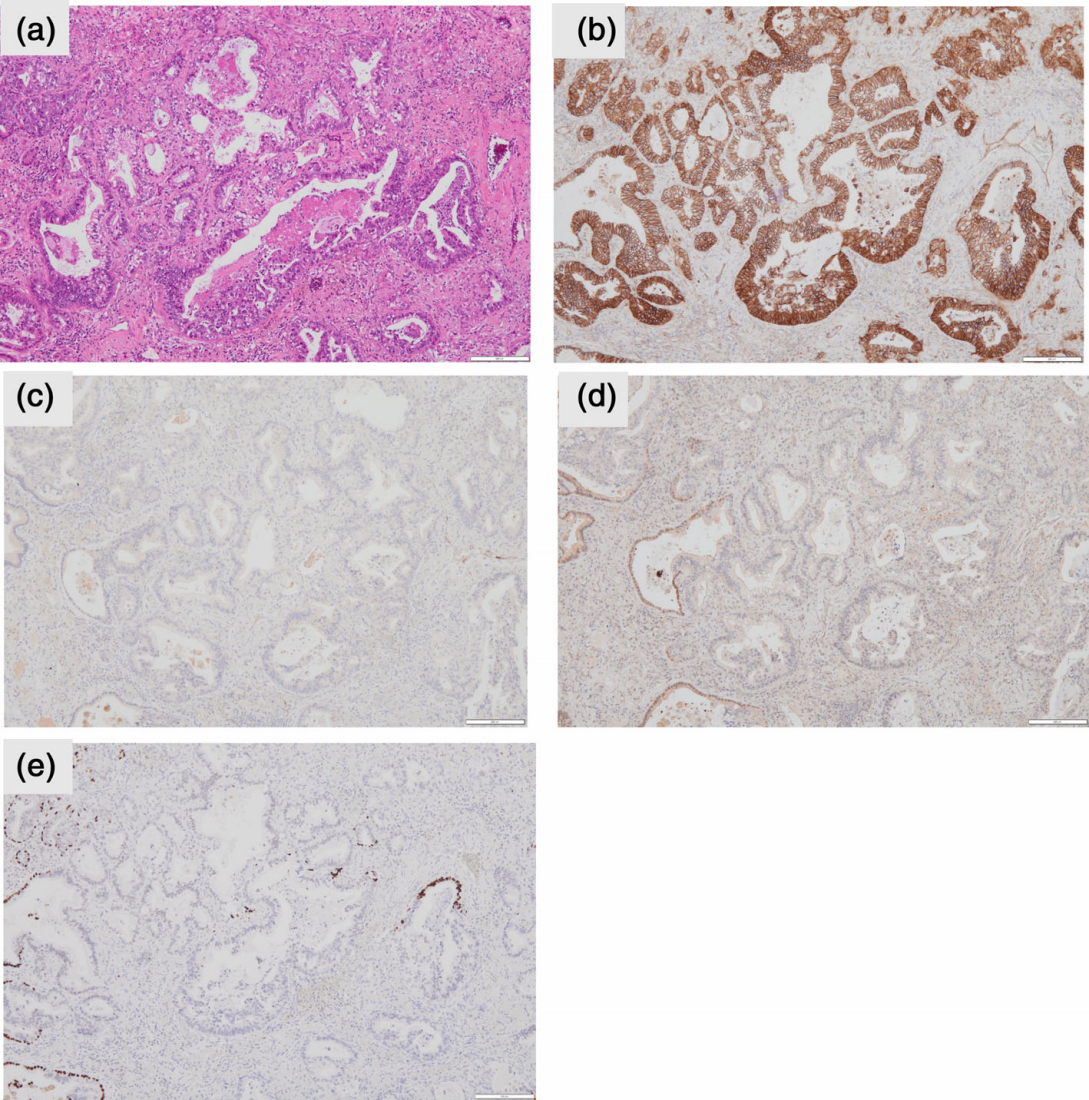
Microscopic findings and special and immunohistochemical stains of high‐grade fetal adenocarcinoma. (a) Hematoxylin–eosin. (b) β‐catenin. (c) Glypican‐3. (d) Sal‐like protein 4. (e) Thyroid transcription factor‐1.

**FIGURE 4:**
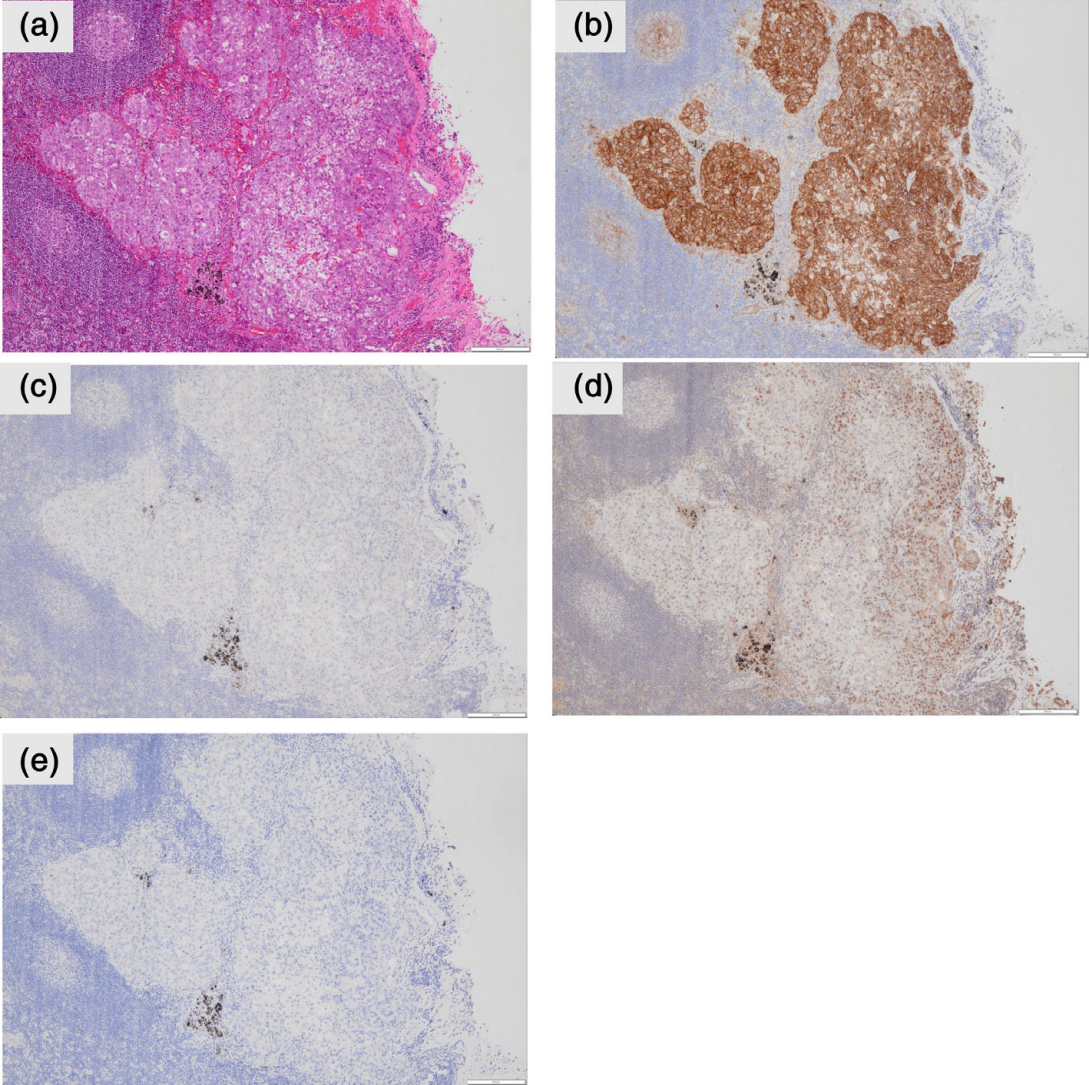
Microscopic finding and special and immunohistochemical stains of lobar bronchial lymph node (#11). (a) Hematoxylin–eosin. (b) β‐catenin. (c) Glypican‐3. (d) Sal‐like protein 4. (e) Thyroid transcription factor‐1.

Immunohistochemical staining of the papillary and lepidic morphologies was positive for the expression of thyroid transcription factor‐1 (TTF‐1) and cytokeratin 7 (CK7) but negative for CD20, caudal‐type homeobox‐2 (CDX‐2), mucoprotein‐2 (MUC2), α‐fetoprotein, sal‐like protein 4 (SALL‐4), and glypican‐3 (Figure 2a,b,d,e). Immunohistochemical staining of the fetal morphology was positive for the expression of CK7, CD20, CDX2, and MUC2 but negative for TTF‐1, α‐fetoprotein, SALL‐4, and glypican‐3 (Figures 2c,f and 3a–e). β‐catenin was only expressed in tumor cell membranes without nuclear/cytoplasmic expression consistent with that observed in H‐FLAC (Figure 3b). The histomorphological and immunohistological features of #11 metastasis were similar to those of H‐FLACs (Figure 4a–e). The main tumor tested with the Oncomine Dx target test had papillary, lepidic, and fetal adenocarcinoma components, but the results were negative. Moreover, the metastatic lymph node tested with the Oncomine Dx target test showed negative results. Furthermore, the main tumor and metastatic lymph node tested negative for programmed cell death 1 ligand 1 (PD‐L1) (Dako, clone 22C3). At the time of this report, the patient is doing well and is in recurrence‐free follow‐up 8 months after surgery.

## DISCUSSION

Fetal adenocarcinoma is subcategorized into low‐ and high‐grade according to the IASLC/ATS/ERS classification of lung adenocarcinoma based on differences in clinicopathological features and prognoses.^3^ H‐FLAC is reportedly more common in men, and almost all patients with H‐FLAC currently smoke or have a history of smoking. Symptoms such as cough and blood sputum were observed in approximately one‐half of patients.^4^ Approximately 40% of H‐FLAC cases are discovered at stage ≥II, and approximately 40% of patients die within 2 years after surgery.^4^ Although our patient had no symptoms, he was a smoker and his clinical stage was II.

In H‐FLAC, components of normal adenocarcinoma are often found in addition to fetal lung‐like components.^2^, ^3^ A mixture of squamous cell carcinoma, large cell neuroendocrine carcinoma, intestinal type adenocarcinoma, and small cell carcinoma has also been reported.^5^ The present case was of a mixed type with papillary lung adenocarcinoma. Immunohistochemically, oncofetal markers that indicate that the fetal‐type component is a fetal component include α‐fetoprotein, glypican‐3, and SALL4. However, >50% of cases are negative for the oncofetal markers,^5^ and in H‐FLAC cases, there is no highly specific immunohistochemical examination. It has been reported that 37% of mixed H‐FLAC tumors were negative for TTF‐1,^6^, ^7^ and β‐catenin was expressed predominantly at the plasma membrane in high‐grade cases.^8^ Our patient's H‐FLAC component and lymph node metastasis were negative for TTF‐1 and positive for β‐catenin in the cell membrane; therefore, H‐FLAC was proven to be the cause of lymph node metastasis. Although there have been several reports of lung cancer with fetal lung adenocarcinoma,^2^, ^3^, ^4^, ^5^, ^9^ this is the first report to demonstrate the histological characteristics of H‐FLAC as the cause of N1 in a patient with lung adenocarcinoma containing a small number of H‐FLAC components. In addition, this is the first study to report results obtained using the Oncomine Dx target test, which were all negative. In addition to the present case, a prior case also showed no PD‐L1 expression. Therefore, even a small amount of H‐FLAC tissue components may be a cause of metastasis. We consider this report extremely useful because it reminds us of the dangers of H‐FLAC.

## AUTHOR CONTRIBUTIONS

Daichi Ishii wrote the manuscript and performed the literature search. Daichi Ishii, Satoshi Aoyama, Wataru Arai, Akihiko Tanaka, Masaru Hasegawa, Takahiro Tsuji, Motoki Sakuraba treated and observed the patient. Motoki Sakuraba supervised the presentation of this case report. All authors read and approved the final manuscript.

## CONFLICT OF INTEREST STATEMENT

The authors declare no conflicts of interest.

## Data Availability

Data sharing is not applicable to this article as no datasets were generated or analyzed for the study.
